# Supplementary material for: Differences in cortical activation patterns during action observation, action execution, and interpersonal synchrony between children with or without autism spectrum disorder (ASD): An fNIRS pilot study
Source: PLoS One. 2020 Oct 29;15(10):e0240301. doi: 10.1371/journal.pone.0240301 (PMC7595285; doi:10.1371/journal.pone.0240301)

S2 Fig. Second to second blocked HbO_2 data per condition and channel for *children with ASD*. Pink vertical line denotes the start of the stimulation period and the data shown to the right of the pink line are the 240 frames across stimulation (10–13 s) and post-stimulation baseline (14–11 s) periods.

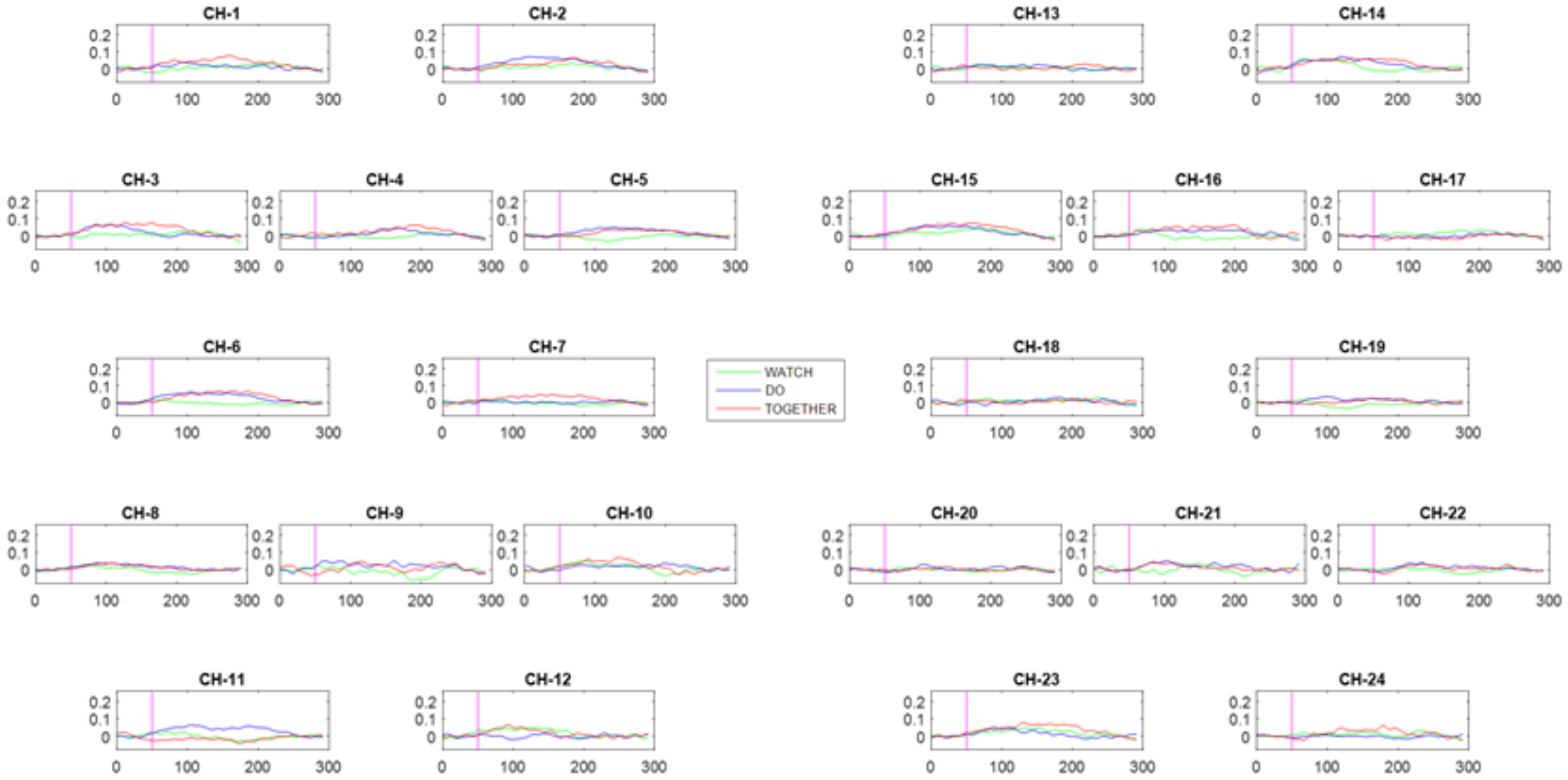

Supplement: S2 Fig — (PDF) [file pone.0240301.s002.pdf]
