# Supplementary material for: Differences in cortical activation patterns during action observation, action execution, and interpersonal synchrony between children with or without autism spectrum disorder (ASD): An fNIRS pilot study
Source: PLoS One. 2020 Oct 29;15(10):e0240301. doi: 10.1371/journal.pone.0240301 (PMC7595285; doi:10.1371/journal.pone.0240301)
Supplement: S1 Table — (PDF) [file pone.0240301.s003.pdf]

S1 Table: Assignment of channels to regions of interest (ROIs) based on spatial registration data. Each channel's position in the MNI coordinate system and an estimate of brain region under the channel are listed. Each channel was modeled as a centroid of a sphere. The percentages represent how much a particular gyrus was within the sphere surrounding the channel. Channels 1 to 12 belonged to the left hemisphere and channels 13 to 24 belong to the right hemisphere. The color-coded channels were considered within a specific ROI (MIFG, IPL, and MSTG) when conducting averaged channel analysis. The bolded channels represented the specific ROIs for channel-specific analysis due to their high coverage of that ROI.

**Side**	**Ch**	**MNI's coordinate system**			**MIFG regions**			**IPL regions**			**MSTG regions**		**Assigned region**
		X	Y	Z	Inferior frontal gyrus	Middle frontal gyrus	Precentral gyrus	Postcentral gyrus	Supramarginal gyrus	Angular gyrus	Middle temporal gyrus	Superior temporal gyrus	
**left**	1	-50.00	7.67	50.67	-	59.9	40.1	-	-	-	-	-	MIFG
	2	-57.00	-23.00	55.00	-	-	-	57.5	42.5	-	-	-	IPL
	**3**	-52.00	24.67	36.67	0.6	**99.4**	-	-	-	-	-	-	MIFG
	4	-61.33	-7.67	41.67	-	-	32.9	66.2	0.8	-	-	-	IPL
	**5**	-64	-37	45.67	-	-	-	-	**(96.2**	**3.8**)	-	-	IPL
	6	-62.33	8.67	26.67	15.6	2.8	81.2	0.3	-	-	-	-	MIFG
	7	-68	-22.67	31.67	-	-	-	10.7	89.3	-	-	-	IPL
	**8**	-59	23.67	10.67	**100**	-	-	-	-	-	-	-	MIFG
	9	-68	-9.33	16.33	-	-	8.2	86.8	2.5	-	-	2.5	Excluded*
	10	-69	-38.67	19.33	-	-	-	-	25.3	-	-	74.7	MSTG
	**11**	-62.33	3.67	-2.33	11.2	-	23.7	1	-	-	-	**65.1**	MSTG
	**12**	-71	-26.33	1.33	-	-	-	-	-	-	**(54.3**	**45.7)**	MSTG
**right**	13	56.00	-25.00	58.00	-	-	-	4.8	95.2	-	-	-	IPL
	14	52.33	5.33	52.33	-	24.0	73.3	2.7	-	-	-	-	MIFG
	**15**	62.67	-41.67	49.33	-	-	-	-	**(45.1**	**54.9)**	-	-	IPL
	16	64.00	-11.33	43.67	-	-	1.1	36.4	62.5	-	-	-	IPL
	**17**	55.00	20.67	36.67	11.7	**66.0**	22.3	-	-	-	-	-	MIFG
	18	70.00	-27.67	33.67	-	-	-	0.5	95.1	4.4	-	-	IPL
	19	66.00	3.67	28.67	-	-	61.4	38.6	-	-	-	-	MIFG
	20	67.00	-45.67	20.67	-	-	-	-	6.5	8.1	29.3	56.0	MSTG
	21	70.00	-13.67	16.33	-	-	-	50.3	3.7	-	-	46.0	Excluded*
	**22**	62.00	18.67	11.33	**42.0**	-	58.0	-	-	-	-	1.6	MIFG
	**23**	73.00	-32.33	2.33	-	-	-	-	-	-	**(62.8**	**37.2)**	MSTG
	**24**	68.00	-1.67	-2.67	-	-	3.5	1.6	-	-	**(6.5**	**88.3)**	MSTG
