# Supplementary material for: Differences in cortical activation patterns during action observation, action execution, and interpersonal synchrony between children with or without autism spectrum disorder (ASD): An fNIRS pilot study
Source: PLoS One. 2020 Oct 29;15(10):e0240301. doi: 10.1371/journal.pone.0240301 (PMC7595285; doi:10.1371/journal.pone.0240301)
Supplement: S2 Table — (PDF) [file pone.0240301.s004.pdf]

S2 Table Significant *p*-values and direction of effects for post-hoc comparisons based on channel-specific ANOVA findings. We conducted a repeated-measures ANOVA and post-hoc analyses for channel-specific regional comparisons. The repeated-measures ANOVA of condition (Watch, Do, Together) x hemisphere (left, right) x channel assignment (MFG, IFG, STG, MTG, IPL) revealed main effects of condition ($F(1.9, 360.4) = 21.8$, $p < 0.001$) and channel ($F(3.5, 668.8) = 7.2$, $p < 0.001$), 2-way interactions of channel x group ($F(3.5, 668.8) = 14.8$, $P < 0.001$), condition x hemisphere ($F(1.9, 361.0) = 8.3$, $p < 0.001$), condition x channel ($F(6.5, 1265.0) = 5.9$, $p < 0.001$), and hemisphere x channel ($F(3.4, 666.6) = 4.5$, $p = 0.002$), 3-way interactions of hemisphere x channel x group interaction ($F(3.4, 666.6) = 6.1$, $p < 0.001$), and condition x hemisphere x channel ($F(7.0, 1352.2) = 4.1$, $p < 0.001$), as well as a four way interaction of condition x hemisphere x channel x group ($F(7.0, 1352.2) = 4.1$, $p < 0.001$) . Note that the results of channel-specific ANOVA are similar to that of the overall regional ANOVA described in the main body of the paper. The results of post-hoc analyses are also similar and are listed in the table below.

Comparison	Significant *p* values	Direction of effect
**Group differences**		
Watch, Left MFG	0.011	ASD > TD [a]
Watch, Right IFG	0.017	TD > ASD [a]
Watch, Right STG	0.003	TD > ASD [a]
Do, Left STG	0.013	TD > ASD [a]
Do, Left MTG	0.028	TD > ASD [b]
Do, Right IFG	0.008	TD > ASD [a]
Do, Right STG	< 0.001	TD > ASD [a]
Do, Right MTG	0.027	TD > ASD [b]
Do, Right IPL	0.001	ASD > TD [a]
Together, Left MFG	0.017	ASD > TD [a]
Together, Left STG	< 0.001	TD > ASD [a]
Together, Right MFG	0.015	TD > ASD [a]
Together, Right IFG	0.009	TD > ASD [a]
Together, Right STG	0.034	TD > ASD [b]
Together, Right IPL	0.006	ASD > TD [a]
**Conditional differences**		
TD, Left MFG	< 0.001	D > W [a]
	< 0.001	T > W [a]
TD, Left IFG	< 0.001	D > W [a]
	0.013	T > W [a]
TD, Left STG	< 0.001	D > W [a]
	0.004	T > W [a]
TD, Left MTG	0.021	T > W [b]
TD, Left IPL	0.008	D > W [a]
	0.014	T > W [a]
TD, Right MFG	< 0.001	T > W [a]
	0.017	T > D [a]
TD, Right IFG	0.015	D > W [a]
TD, Right MTG	0.017	T > D [a]
TD, Right IPL	0.002	T > W [a]
	0.003	T > D [a]
ASD, Left MFG	0.003	D > W [a]
	< 0.001	T > W [a]
ASD, Left STG	0.024	D > W [b]
	0.020	W > T [b]

	< 0.001	D > T [a]
ASD, Left, IPL	< 0.001	D > W [a]
	< 0.001	T > W [a]
ASD, Right MTG	0.016	T > W [a]
	0.038	T > D [b]
ASD, Right IPL	0.009	D > W [a]
	< 0.001	T > W [a]
**Hemispheric differences**		
TD, Do, MTG	0.025	L > R [b]
TD, Do, MTG	0.006	L > R [a]
TD, Do. IPL	0.045	L > R [b]
ASD, Watch, IPL	0.031	R > L [b]
ASD, Do, MFG	< 0.001	L > R [a]
ASD, Do, MSTS	< 0.001	L > R [a]
ASD, Do, MTG	0.048	R > L [b]
ASD, Together, MFG	< 0.001	L > R [a]
ASD, Together, STG	0.009	R > L [a]
ASD, Together, IPL	0.012	R > L [b]
