# Supplementary material for: Differences in cortical activation patterns during action observation, action execution, and interpersonal synchrony between children with or without autism spectrum disorder (ASD): An fNIRS pilot study
Source: PLoS One. 2020 Oct 29;15(10):e0240301. doi: 10.1371/journal.pone.0240301 (PMC7595285; doi:10.1371/journal.pone.0240301)
Supplement: S1 File — (ZIP) [file pone.0240301.s005.zip › SupportingInformation_2/4_PONE-D-19-34634R2_SupportingInformation.docx]

**S1 Fig.** Second to second blocked HbO_2_ data per condition and channel for *TD children*. Pink vertical line denotes the start of the stimulation period and the data shown to the right of the pink line are the 240 frames across stimulation (10–13 s) and post-stimulation baseline (14–11 s) periods.

**
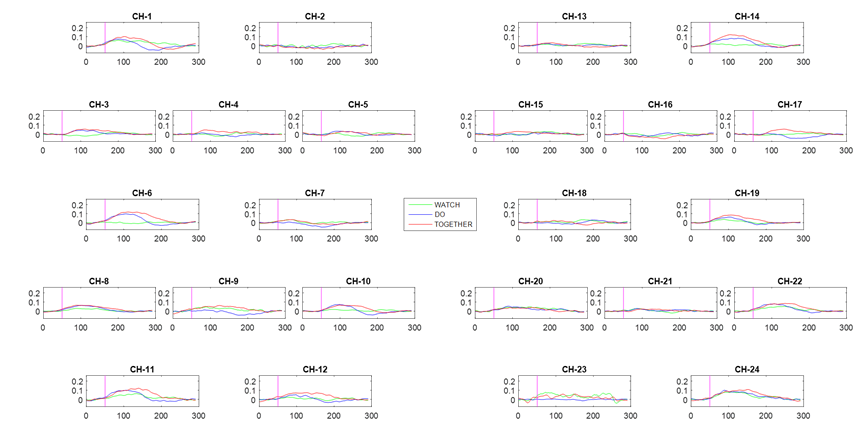
**

**S2 Fig.** Second to second blocked HbO_2_ data per condition and channel for *children with ASD*. Pink vertical line denotes the start of the stimulation period and the data shown to the right of the pink line are the 240 frames across stimulation (10–13 s) and post-stimulation baseline (14–11 s) periods.


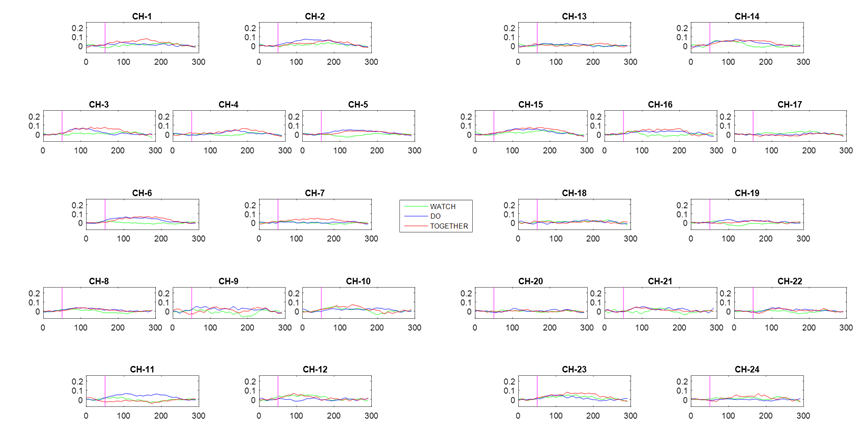


**S1 Table** shows channel assignments based on our spatial registration approach. For each channel, the spatial location in MNI’s coordinate system and the probability of covering different brain regions are shown. Channels 1 to 12 belonged to the left hemisphere and channels 13 to 24 belong to the right hemisphere. The color-coded channels were considered within a specific ROI (MIFG, IPL, and MSTG) when conducting averaged channel analysis. The bolded channels represent a specific ROI (MIFG, IPL and MSTG) for channel-specific analysis due to their high coverage of that ROI.

| Side | Ch | MNI’s coordinate system | | | MIFG regions | | | IPL regions | | | MSTG regions | | Assigned region |
| --- | --- | --- | --- | --- | --- | --- | --- | --- | --- | --- | --- | --- | --- |
|  |  | X | Y | Z | Inferior frontal gyrus | Middle frontal gyrus | Precentral gyrus | Postcentral gyrus | Supramarginal gyrus | Angular gyrus | Middle temporal gyrus | Superior temporal gyrus |  |
| left | 1 | -50.00 | 7.67 | 50.67 | - | 59.9 | 40.1 | - | - | - | - | - | MIFG |
|  | 2 | -57.00 | -23.00 | 55.00 | - | - | - | 57.5 | 42.5 | - | - | - | IPL |
|  | **3** | -52.00 | 24.67 | 36.67 | 0.6 | **99.4** | - | - | - | - | - | - | MIFG |
|  | 4 | -61.33 | -7.67 | 41.67 | - | - | 32.9 | 66.2 | 0.8 | - | - | - | IPL |
|  | **5** | -64 | -37 | 45.67 | - | - | - | - | **(96.2** | **3.8**) | - | - | IPL |
|  | 6 | -62.33 | 8.67 | 26.67 | 15.6 | 2.8 | 81.2 | 0.3 | - | - | - | - | MIFG |
|  | 7 | -68 | -22.67 | 31.67 | - | - | - | 10.7 | 89.3 | - | - | - | IPL |
|  | **8** | -59 | 23.67 | 10.67 | **100** | - | - | - | - | - | - | - | MIFG |
|  | 9 | -68 | -9.33 | 16.33 | - | - | 8.2 | 86.8 | 2.5 | - | - | 2.5 | Excluded* |
|  | 10 | -69 | -38.67 | 19.33 | - | - | - | - | 25.3 | - | - | 74.7 | MSTG |
|  | **11** | -62.33 | 3.67 | -2.33 | 11.2 | - | 23.7 | 1 | - | - | - | **65.1** | MSTG |
|  | **12** | -71 | -26.33 | 1.33 | - | - | - | - | - | - | **(54.3** | **45.7)** | MSTG |
| right | 13 | 56.00 | -25.00 | 58.00 | - | - | - | 4.8 | 95.2 | - | - | - | IPL |
|  | 14 | 52.33 | 5.33 | 52.33 | - | 24.0 | 73.3 | 2.7 | - | - | - | - | MIFG |
|  | **15** | 62.67 | -41.67 | 49.33 | - | - | - | - | **(45.1** | **54.9)** | - | - | IPL |
|  | 16 | 64.00 | -11.33 | 43.67 | - | - | 1.1 | 36.4 | 62.5 | - | - | - | IPL |
|  | **17** | 55.00 | 20.67 | 36.67 | 11.7 | **66.0** | 22.3 | - | - | - | - | - | MIFG |
|  | 18 | 70.00 | -27.67 | 33.67 | - | - | - | 0.5 | 95.1 | 4.4 | - | - | IPL |
|  | 19 | 66.00 | 3.67 | 28.67 | - | - | 61.4 | 38.6 | - | - | - | - | MIFG |
|  | 20 | 67.00 | -45.67 | 20.67 | - | - | - | - | 6.5 | 8.1 | 29.3 | 56.0 | MSTG |
|  | 21 | 70.00 | -13.67 | 16.33 | - | - | - | 50.3 | 3.7 | - | - | 46.0 | Excluded* |
|  | **22** | 62.00 | 18.67 | 11.33 | **42.0** | - | 58.0 | - | - | - | - | 1.6 | MIFG |
|  | **23** | 73.00 | -32.33 | 2.33 | - | - | - | - | - | - | **(62.8** | **37.2)** | MSTG |
|  | **24** | 68.00 | -1.67 | -2.67 | - | - | 3.5 | 1.6 | - | - | **(6.5** | **88.3)** | MSTG |

**S2 Table** Significant *p*-values and direction of effects for post-hoc comparisons based on channel-specific ANOVA findings. We conducted a repeated-measures ANOVA and post-hoc analyses for channel-specific regional comparisons. The repeated-measures ANOVA of condition (Watch, Do, Together) x hemisphere (left, right) x channel assignment (MFG, IFG, STG, MTG, IPL) revealed main effects of condition (F(1.9, 360.4) = 21.8, *p* < 0.001) and channel (F(3.5, 668.8) = 7.2, *p* < 0.001), 2-way interactions of channel x group (F(3.5, 668.8) = 14.8, *P* < 0.001), condition x hemisphere (F(1.9, 361.0) = 8.3, *p* < 0.001), condition x channel (F(6.5, 1265.0) = 5.9, *p* < 0.001), and hemisphere x channel (F(3.4, 666.6) = 4.5, *p* = 0.002), 3-way interactions of hemisphere x channel x group interaction (F(3.4, 666.6) = 6.1, *p* < 0.001), and condition x hemisphere x channel (F(7.0, 1352.2) = 4.1, *p* < 0.001), as well as a four way interaction of condition x hemisphere x channel x group (F(7.0, 1352.2) = 4.1, *p* < 0.001) . Note that the results of channel-specific ANOVA are similar to that of the overall regional ANOVA described in the main body of the paper. The results of post-hoc analyses are also similar and are listed in the table below.

| **Comparison** | **Significant *p* values** | **Direction of effect** |
| --- | --- | --- |
| **Group differences** | | |
| Watch, Left MFG  Watch, Right IFG  Watch, Right STG | 0.011  0.017  0.003 | ASD > TD ^a^  TD > ASD ^a^  TD > ASD ^a^ |
| Do, Left STG  Do, Left MTG  Do, Right IFG  Do, Right STG  Do, Right MTG  Do, Right IPL | 0.013  0.028  0.008  < 0.001  0.027  0.001 | TD > ASD ^a^  TD > ASD ^b^  TD > ASD ^a^  TD > ASD ^a^  TD > ASD ^b^  ASD > TD ^a^ |
| Together, Left MFG  Together, Left STG  Together, Right MFG  Together, Right IFG  Together, Right STG  Together, Right IPL | 0.017  < 0.001  0.015  0.009  0.034  0.006 | ASD > TD ^a^  TD > ASD ^a^  TD > ASD ^a^  TD > ASD ^a^  TD > ASD ^b^  ASD > TD ^a^ |
| **Conditional differences** | | |
| TD, Left MFG  TD, Left IFG  TD, Left STG  TD, Left MTG  TD, Left IPL  TD, Right MFG  TD, Right IFG  TD, Right MTG  TD, Right IPL | < 0.001  < 0.001  < 0.001  0.013  < 0.001  0.004  0.021  0.008  0.014  < 0.001  0.017  0.015  0.017  0.002  0.003 | D > W ^a^  T > W ^a^  D > W ^a^  T > W ^a^  D > W ^a^  T > W ^a^  T > W ^b^  D > W ^a^  T > W ^a^  T > W ^a^  T > D ^a^  D > W ^a^  T > D ^a^  T > W ^a^  T > D ^a^ |
| ASD, Left MFG  ASD, Left STG  ASD, Left, IPL  ASD, Right MTG    ASD, Right IPL | 0.003  < 0.001  0.024  0.020  < 0.001  < 0.001  < 0.001  0.016  0.038  0.009  < 0.001 | D > W ^a^  T > W ^a^  D > W ^b^  W > T ^b^  D > T ^a^  D > W ^a^  T > W ^a^  T > W ^a^  T > D ^b^  D > W ^a^  T > W ^a^ |
| **Hemispheric differences** | | |
| TD, Do, MTG  TD, Do, MTG  TD, Do. IPL | 0.025  0.006  0.045 | L > R ^b^  L > R ^a^  L > R ^b^ |
| ASD, Watch, IPL  ASD, Do, MFG  ASD, Do, MSTS  ASD, Do, MTG  ASD, Together, MFG  ASD, Together, STG  ASD, Together, IPL | 0.031  < 0.001  < 0.001  0.048  < 0.001  0.009  0.012 | R > L ^b^  L > R ^a^  L > R ^a^  R > L ^b^  L > R ^a^  R > L ^a^  R > L ^b^ |
