# Supplementary material for: Differences in cortical activation patterns during action observation, action execution, and interpersonal synchrony between children with or without autism spectrum disorder (ASD): An fNIRS pilot study
Source: PLoS One. 2020 Oct 29;15(10):e0240301. doi: 10.1371/journal.pone.0240301 (PMC7595285; doi:10.1371/journal.pone.0240301)
Supplement: Attachment — Submitted filename: 0B_ResponsetoReviewers_IPS_TDvsASD_091220.docx [file pone.0240301.s007.docx]

Thank you for all your efforts in reviewing and improving this manuscript! Here we address the last few reviewer concerns. We have further modified the manuscript based on their comments. Each edit within the manuscript is highlighted using track changes. Changes within the manuscript are listed here by providing the page # and line #.

**Reviewer 2, Comment 1:** I appreciate the switch from subgroup analysis to correlations but I do not see any mention of control for multiple comparisons in this section. Have the authors considered multiple comparisons in their correlation analysis?

**Response:** Yes, we used the False Discovery Rate (FDR) method for fNIRS-based analysis proposed by Singh and Dan (2006) to account for multiple comparisons during post-hoc testing as well as correlational analyses. We specifically used the Benjamin-Hochberg method wherein unadjusted *p*-values are rank ordered from low to high. *p*-value thresholds were determined by multiplying 0.05 with the ratio of the unadjusted *p*-value rank to the total # of comparisons (*p*-threshold for ith comparison = 0.05 x i/n; where n=total # of comparisons). Statistical significance is declared if the unadjusted *p*-value is less than the *p*-value threshold. We have added this clarification descriptions within the statistical analysis section (Page 20, Line 426-427).

**Comment 2:** The authors have addressed my comments but I still do not have a sense of how this cohort compares to cohorts in fMRI studies of ASD. Could the authors provide a brief summary of severity across fMRI studies (or at least the ones they cite) and how that compares to the present study’s cohort? I also do not see any corresponding changes in the manuscript. I think this information is important to include for all readers of the manuscript.

**Response:** The current fNIRS study was able to include children with lower IQ and greater ASD severity assessed using the ADOS compared to participants in most fMRI studies. In a meta-analysis of fMRI findings in individuals with ASD (Philip et al., 2012), among the 23 studies that reported IQ in children with ASD, only 1 study included children with lower IQ (Mean IQ= 76.8, Okem et al., 2000). The rest of the studies had a sample mean IQ ranging from 90.7 to 116.0 (SD ranged from 12.3 to 27.7, Philip et al., 2012). Similarly, in a more recent meta-analysis of fMRI studies of ASD during observation and imitation tasks, the IQ scores were much higher than that of the present study (our mean IQ was 79.6 ± 25.4). Specifically, the sample mean IQ ranged from 93.3 to 124.8 for the 13 studies included in the analysis.

The ADOS scores for some of the fMRI studies were lower (indicating less severe ASD) than the present study (Dougherty et al., 2016: mean ADOS total score = 11.7, SD = 3.5; Dona et al., 2017: mean ADOS comparison score = 6.5, SD = 2.2; present study: mean ADOS total score = 18.2, SD = 1.9; ADOS comparison score: 8.4, SD = 6.7, Table 1). We have added this information comparing fMRI studies and the present study within the discussion session (Page 27, line 543-554).

**Reviewer 3, Comment 1:** The new text in lines 629-635 does a better job of accounting for the potential of perceptual and motor issues both being salient. However, it is still worth a bit of emphasis, and maybe could be covered by adding at the end of that paragraph something like ‘One cannot exclude the possibility that, due to impaired perception of the salience of the action information from the partner, that the input is decreased upstream of the IFG and MFG (from higher order perceptual inputs), contributing to hypoactivation.’ As an obvious example for demonstration of this point, someone with cortical blindness would have hypoactivation in these regions in their effort to do these tasks, and impaired performance, obviously, but not resulting from an executive functioning problem. This would also tie in nicely with the new text in the subsequent paragraph.

**Response:** Thank you for suggesting the possibility of reduced perception of action information from partners leading to hypoactivation in the MFG and IFG regions. We have added your suggested phrasing within the discussion session (Page 31, line 648-649).

Once again we truly appreciate all the efforts in reviewing this manuscript. We look forward to hearing from you.
